# AGHmatrix: genetic relationship matrices in R

**DOI:** 10.1093/bioinformatics/btad445

**Published:** 2023-07-20

**Authors:** Rodrigo R Amadeu, Antonio Augusto F Garcia, Patricio R Munoz, Luís Felipe V Ferrão

**Affiliations:** Bayer U.S.—Crop Science, Chesterfield, MO, United States; Departamento de Genética, Escola Superior de Agricultura Luiz de Queiroz, Universidade de São Paulo, Piracicaba, SP, Brazil; Blueberry, Breeding and Genomics Lab, Horticultural Sciences Department, University of Florida, Gainesville, FL 32611, United States; Blueberry, Breeding and Genomics Lab, Horticultural Sciences Department, University of Florida, Gainesville, FL 32611, United States

## Abstract

**Motivation:**

The resemble between relatives computed from pedigree and genomic data is an important resource for geneticists and ecologists, who are interested in understanding how genes influence phenotypic variation, fitness adaptation, and population dynamics.

**Results:**

The AGHmatrix software is an R package focused on the construction of pedigree (**A** matrix) and/or molecular markers (**G** matrix), with the possibility of building a combined matrix of pedigree corrected by molecular markers (**H** matrix). Designed to estimate the relationships for any ploidy level, the software also includes auxiliary functions related to filtering molecular markers, and checks pedigree errors in large data sets. After computing the relationship matrices, results from the AGHmatrix can be used in different contexts, including on prediction of (genomic) estimated breeding values and genome-wide association studies.

**Availability and implementation:**

AGHmatrix v2.1.0 is available under GPL-3 license in CRAN at https://cran.r-project.org/web/packages/AGHmatrix/index.html and also in GitHub at https://github.com/rramadeu/AGHmatrix. It has a comprehensive tutorial, and it follows with real data examples.

## 1 Introduction

Quantitative genetics is a discipline with a distinguished history in multiple biological fields. Geneticists and ecologists have relied on genetic parameters to understand how genes influence phenotypic variation, fitness adaptation, and population dynamics (Wilson *et al.* 2010, Walsh and Lynch 2018). Breeders, on the other hand, are more interested in predicting empirical breeding values to design crosses and select the best progeny. For these cases, a fundamental premise underlying any genetic analyses is that individuals in a population are not independent and can be genetically connected by their actual (recorded) or estimated pedigree. The resemblance between relatives can provide the means to make inference about the inheritance of complex traits, even without explicit knowledge of the genes involved, and provide the basis for approaches such as genomic selection (Wilson *et al.* 2010). As a practical result, the phenotypic variance associated with complex traits can be decomposed into their genetic action portions—additive, dominance, and epistasis sources of variations—providing a range of theoretical and empirical tools to answer important biological questions.

Important genetic analyses rely on the estimation of resemblance between relatives. For example, multivariate analyses (i.e. principal component analysis) are commonly employed to describe the genetic diversity of a population, while linear mixed models are used to predict breeding values and estimate variance components partition. In common, most of these analyses have been carried out in R, one of the most popular statistical programming environments used by breeders and biometricians. Despite its popularity, computing relationship matrices including (i) pedigree records and molecular information, (ii) for diploid and polyploid species, and (iii) using different parametrizations (additive, dominance, and epistasis) have not been addressed in the same package using a unique framework. To overcome it, we designed the AGHmatrix R package to compute relationship matrices for a range of scenarios, providing an efficient software to large-scale data sets.

## 2 Package description

AGHmatrix software is an R package to build genetic relationship matrices. The software was initially released for pedigree analyses in outcrossing plants (Amadeu *et al.* 2016). At that time, it was one of the first R package to estimate pedigree relatedness between any two organisms in plants, while considering ploidy level and ignoring entity gender. After its initial release, we maintained and upgraded its functionalities, with the possibility to compute different relationship matrices using molecular information and multiple gene actions. Today, it is an established R package on the breeding community with documented applications in a range of breeding systems. Our goal is to fully present its current capability and upgrades since the initial release.

The software is focused on the construction of pedigree (**A** matrix) and/or molecular markers (**G** matrix) with the possibility of building a combined matrix of pedigree corrected by molecular markers (**H** matrix). The package works for diploid and autopolyploid species, fitting 15 different relationship matrices well-reported in the plant and animal literature (Table 1). The package is structured around three functions (Amatrix, Gmatrix, and Hmatrix) depending on the type of information available for computing relationship.

**Table 1.** Different ways to compute relationship matrices implemented in AGHmatrix software.

Source	Ploidy	Parametrization	Reference	Function	Argument
Pedigree (A)	Diploid	Additive	Henderson (1976)	Amatrix()	Default
Pedigree (A)	Diploid	Nonadditive	Cockerham (1954)	Amatrix()	Dominance = TRUE
Pedigree (A)	Polyploid	Additive	Kerr (2012)	Amatrix()	Ploidy = XX, w = YY
Pedigree (A)	Polyploid	Additive	Slater et al. (2014)	Amatrix()	Ploidy = XX, slater = TRUE

Genomic (G)	Diploid	Additive	Yang (2010)	Gmatrix()	Method = “Yang”
Genomic (G)	Diploid	Additive	VanRaden (2008)	Gmatrix()	Method = “VanRaden”
Genomic (G)	Diploid	Additive	Liu (2020)	Gmatrix()	Method = “Liu”
Genomic (G)	Diploid	Nonadditive	Su (2012)	Gmatrix()	Method = “Su”
Genomic (G)	Diploid	Nonadditive	Vitezica (2013)	Gmatrix()	Method = “Vitezica”
Genomic (G)	Polyploid	Additive	Slater (2016)	Gmatrix()	Ploidy = XX, method = “Slater”
Genomic (G)	Polyploid	Additive	VanRaden (2008)	Gmatrix()	Ploidy = XX, method = “VanRaden”
Genomic (G)	Polyploid	Nonadditive	Endelman (2018)	Gmatrix()	Ploidy = XX, method = “Endelman”
Genomic (G)	Polyploid	Ratio	de Bem Oliveira (2019)	Gmatrix()	Ploidy = XX, method = “VanRaden”, ratio = TRUE

Hybrid (H)	Any ploidy	Additive	Martini (2018)	Hmatrix()	Method = “Martini”
Hybrid (H)	Any ploidy	Additive	Munoz (2014)	Hmatrix()	Method = “Munoz”

XX is any even-ploidy number, and YY is the expected double-reduction fraction (between 0 and 1).

### 2.1. Pedigree records (Amatrix)

The Amatrix function computes additive relationship matrices (**A**) after reading pedigree tables recorded in a three-column format, where the first column contains the genotype ID, followed by Parent 1 and Parent 2 IDs. Parent 1 and 2 assignment is arbitrary with no sex distinction, key factor for monoecious breeding. Matrices are built based on the recursive method presented in Mrode (2014) and described by Henderson (1976). The same method is also implemented for higher ploidies (even-ploidy) that considers double reduction as detailed in Kerr *et al.* (2012). Internally the algorithm works in two stages. First, it preprocesses the pedigree, and individuals are numerated from 1 to *N*, where *N* is the total number of individuals in the pedigree. Individuals IDs are checked for missing values and for the correct chronological order (i.e. if the parents of a given individual are located before to this individual in the pedigree data set). If this order is not followed, the algorithm performs the necessary changes to correct it iteratively. After this preprocessing, the second stage relies on matrix algebra to efficiently build A. There is also the possibility to use a prebuilt A and to add new pedigree information (expandAmatrix function), so there is no need to rebuild the A every time new crosses are planned or made.

There is also the possibility of assuming a nondeterminist pedigree. AmatrixPolyCross function was designed considering the following scenario: a mating design in which equally possible parents are present, for example, an offspring harvested in bulk derived from the mating of a group of parents (bulk breeding). In this case, all seeds will have the same expected relatedness with all the possible parents (1/3) while assuming no inbreeding. Within this function, it is also possible to fix a given parent and mimics the case where the mother is known, and we have equally possible pollen donors (e.g. polycross in sugarcane and other grasses).

### 2.2 Genomic information (Gmatrix)

A second function, the Gmatrix, handles the molecular-marker matrix and builds realized relationship matrices (**G**) using different approaches (Table 1). Molecular-marker data should be organized in a matrix format (individuals in rows and markers in columns) in which the allele dosages are provided. For example, diploid organisms should have a maximum of three genotypic classes classified as 0, 1, and 2, depending on the number of reference alleles. Similarly, tetraploid species contain a maximum of five classes with nulliplex, simplex, duplex, triplex, and quadruplex codified as 0, 1, 2, 3, and 4, respectively. Higher ploidy numbers are also allowed. When importing the data as a matrix, the software can filter the molecular information by excluding markers depending on the minimal allele frequency (MAF), missing data (call rate), monomorphic markers, and observed heterozygosity. Missing data can be imputed using the mean or the mode.

For diploids, the Gmatrix function was implemented to construct additive relationship methods as proposed by Yang *et al.* (2010) and VanRaden (2008). For dominance, diploid matrices are built following the approaches described either by Su *et al.* (2012) or Vitezica *et al.* (2013). For polyploids, more diverse methods are currently implemented in the software, representing the higher complexity of polyploid species. Therefore, the function Gmatrix can be used to construct: (i) the additive relationship based on VanRaden (2008) and extended by Ashraf *et al.* (2016), as described by de Bem Oliveira *et al.* (2019); (ii) the full-autopolyploid including additive and nonadditive model as Equations 8 and 9 described in Slater *et al.* (2016); (iii) the pseudo-diploid model as Equations 5–7 reported in Slater *et al.* (2016); and (iv) the digenic-dominant model based on Endelman *et al.* (2018). There is also an option to build weighted relationship matrices (Su *et al.* 2014, Liu *et al.* 2020).

An important extension in the Gmatrix function is the possibility of computing relationship matrices when markers are scored continuously. The argument ratio = TRUE allows values ranging from 0 to 1, which is useful (i) when genotypic probabilities are used as marker input, (ii) genotypic classes are represented as the count of alternative (or reference) alleles over the total read depth for each individual-marker combination (GBS-like technique), (iii) mixed-ploidy populations are provided and a proxy for the additive matrix should be computed, and (iv) for family-pool genotypes (as in Ashraf *et al.* 2016).

### 2.3 Combined relationship matrix (Hmatrix)

The last main function implemented in the package is the Hmatrix, which was primarily inspired in animal breeding studies where **G** and **A** matrices are combined to compute breeding values based on single-step genome evaluation (Misztal *et al.* 2009). First both the **A** and **G** matrices are computed separately (as explained above). To compute the **H** matrix, two methods are implemented. The approach described by Munoz *et al.* (2014) shrinks the **G** matrix toward the **A** matrix scaling the molecular relatedness by each relationship class. And also, the approach described by Martini *et al.* (2018), a modified version from Legarra *et al.* (2009) in which **A** and **G** matrices are combined using scaling factors, ultimately weighing the importance of pedigree and molecular information when both data are combined. For genomic selection, a valid approach to select the best scaling factor would be testing a grid of values in a cross-validation scheme and checking the impact on predictive ability. The weights can also be managed with the target to build a positive-definite (invertible) **H** matrix.

### 2.4 Other functions

The AGHmatrix also contains supplementary functions that can help biometricians and breeders organize their pedigree information, filter SNP information, and export the results in different formats. For example, the snp.check function can exclude molecular markers depending on the number of missing data (call rate), MAF, heterozygosity (important in the case of breeding lines) and if genetic variants are monomorphic. Simple imputation methods based on the mode or the mean are also available in the same function.

## 3 Application

After computing the relationship matrices, results can be directly consumed as a diversity metric (e.g., to understand population structure, to control for inbreeding in selection and mating allocations). They can also be used in a different context. A straightforward implementation is in the so-called “Animal Model” (or ABLUP), which is a mixed model in which breeding values (or the genetic merit) are included as an explanatory variable for a phenotypic trait of interest. Assuming that genetic merits are random effects, the nonindependence between individuals from the same population is accounted for using pedigree information. A simple expansion of this idea is the inclusion of genomic relationship matrices (GBLUP), which makes results applied to genomic selection studies. Various software and packages can read external relationship matrices for prediction breeding values, including asreml-R (Butler *et al.* 2017) and rrBLUP (Endelman 2011). Another important application is on genome-wide association studies (GWAS) when using the **Q **+** K** method. While the effects of population stratification and hidden relatedness are an important source of spurious associations, using a polygenic term (**G** matrix) for controlling population structure is a crucial element in GWAS models. Some packages, including the GWASpoly (Rosyara *et al.* 2016), can include external (diploid and polyploid) matrices for correcting eventual sample structure.

**Figure 1.**
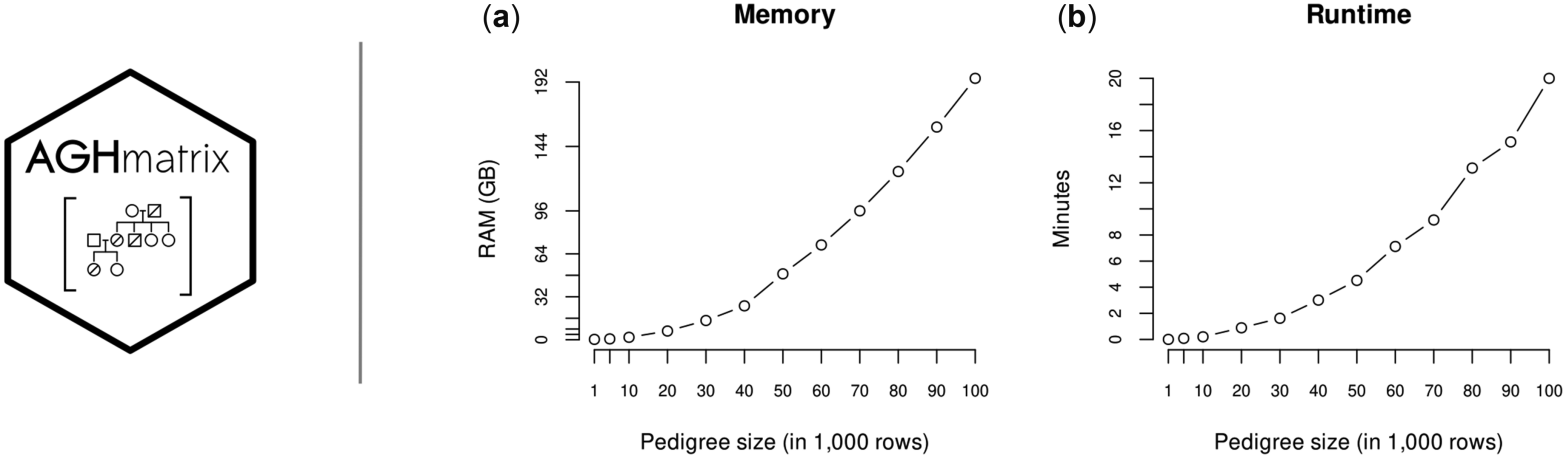
(a) RAM usage at the peak and (b) time to build A matrix for different sizes of pedigree

Finally, we reported some benchmarks for using AGHmatrix when computing large pedigree files (Fig. 1). The package uses a small memory and computational time profiling. The required RAM was computed based on the peak of the process for different pedigree sizes (based on/usr/bin/time-v output). The time profiling was done using AMD Milan 2.95 GHz, so it might be an underestimated value compared to lower-speed processors. Numerator relationship matrices for pedigrees with <20 000 rows can be built with low-specs user-end machines (<8 GB RAM).
